# Risk of contralateral second primary breast cancer according to hormone receptor status in Germany

**DOI:** 10.1186/s13058-014-0452-4

**Published:** 2014-10-03

**Authors:** Carsten Rusner, Katharina Wolf, Ulrike Bandemer-Greulich, Jutta Engel, Christa Stegmaier, Bernd Holleczek, Gabriele Schubert-Fritschle, Anett Tillack, Andreas Stang

**Affiliations:** Institute of Clinical Epidemiology, Medical Faculty, Martin-Luther-University of Halle-Wittenberg, Magdeburger Strasse 8, 06097 Halle (Saale), Germany; Cancer Registry of the Federal State of Brandenburg, Müllroser Chaussee 7, 15236 Frankfurt (Oder), Germany; Munich Cancer Registry of the Munich Cancer Centre, Clinic Großhadern/IBE, Ludwig-Maximilians-University Munich, Marchioninistrasse 15, 81377 Munich, Germany; Saarland Cancer Registry, Präsident-Baltz-Strasse 5, 66119 Saarbrücken, Germany; Department of Epidemiology, School of Public Health, Boston University, 715 Albany Street, Boston, MA 02118 USA

## Abstract

**Introduction:**

Hormone receptor (HR) status has become an established target in treatment strategies of breast cancer. Population-based estimates of contralateral breast cancer (CBC) incidence by HR subtype in particular are limited. The aim of this study was to provide detailed data on CBC incidence for Germany.

**Methods:**

Invasive breast cancer data were extracted on 49,804 women yielding 594 second primaries from the cancer registries of the Federal States of Brandenburg and Saarland and the area of Munich for the period from 1998 to 2007. Multiple imputation was used on missing values for HR status. We estimated standardized incidence ratios (SIRs) with 95% confidence intervals (95%CIs).

**Results:**

SIR estimates of CBC among women diagnosed with an invasive first primary breast cancer (FBC) of any HR subtype ranged from 1.0 to 1.5 in the three registries. Pooling three registries’ data, the SIR of HR-positive CBC was 0.7 (95%CI: 0.6 to 0.8) among women with HR-positive FBC. For those women with HR-negative FBC, the SIR of HR-negative CBC was 8.9 (95%CI: 7.1 to 11.1). Among women with FBC diagnosed before the age of 50 years, incidence of CBC was increased, especially for HR-negative FBC (SIR: 9.2; 95%CI: 7.1 to 11.9).

**Conclusions:**

HR status of the first primary and age at first diagnosis is relevant for predicting risk of CBC. Particularly, patients with HR-negative FBC had elevated risks.

**Electronic supplementary material:**

The online version of this article (doi:10.1186/s13058-014-0452-4) contains supplementary material, which is available to authorized users.

## Introduction

Prognosis of breast cancer has improved over the recent decades by progress in diagnosis and treatment [1]. However, patients with breast cancer have an increased risk of developing a new primary breast cancer in the contralateral breast. Family history of breast cancer, early age at diagnosis, characteristics of the first primary (for example lobular histology and stage) and mutations in specific genes, including BRCA1, BRCA2 and CHEK2, are considered as risk factors [2,3]. Hormone receptor (HR) status of breast cancer is also a relevant factor, in particular with regard to treatment decisions and further prognosis. Treatment strategies of radiotherapy and chemotherapy after surgery of HR-positive breast cancer are followed by an adjuvant hormonal treatment. Hormone treatment with tamoxifen or aromatase inhibitor anastrozole for reducing the risk of contralateral breast cancer (CBC) is well known. A meta-analysis of 55 randomized trials found that use of tamoxifen for five years reduces the risk of CBC by 47% [4]. There is some evidence that tamoxifen treatment may increase the risk of HR-negative CBC [5].

Only few population-based reports showed the impact of HR subtype in invasive first primary breast cancers (FBC) on risk of CBC. Three previously published studies reported an increased risk of HR-negative CBC after HR-negative FBC. Results of developing CBC after HR-positive FBC were contradictory [6-8].

The aim of our study was to provide detailed estimates of CBC incidence according to HR status of FBC using data from population-based cancer registries in Germany. This study also focused on mixed HR status, which has not been presented by previous studies.

## Methods

Female patients diagnosed with FBC were identified in the population-based cancer registries of the Federal States of Saarland and Brandenburg and in the Munich Cancer Registry for the period 1998 to 2007. Ethical approval for this study was not required because we used anonymized data of cancer registries for scientific purposes according to Good Practice for Secondary Data Analysis [9].

The cancer registry of the Federal State of Brandenburg (BB) was established in 1993 and comprises five hospital-based cancer registries covering the entire territory of the Federal State. The Munich Cancer Registry (MCR) was established in 1978 and routinely records data for all cancer patients treated in Munich and the surrounding area. It receives clinical data from 73 hospitals and several hundred doctors in private practice. The Saarland Cancer Registry (SL) is a population-based cancer registry and covers the entire territory of the Federal State of Saarland. The registry was established in 1967. The three registries comprise a population of 6.9 million in total (BB: 2.6, MCR: 3.3, SL: 1.1 million). BB, MCR and SL have been involved in several regional, national and international research collaborations [10]. The registries provide cancer incidence data with an estimated completeness of 90% and more [11]. However, the completeness of HR status registration was too low for a meaningful data analysis until 1998 because German cancer registries do not routinely collect or receive data of hormone receptor status. A special data collection effort from available pathology reports in all three registries allowed us to obtain HR status information for the specified years of 1998 to 2007. For the remaining cases with missing information on HR status, we re-contacted the reporting pathologists.

Invasive breast cancers were coded as C50 according to the 10th edition of the International Classification of Diseases, respectively (ICD-10) [12]. For additional analyses, we defined four major histologic groups of breast cancer: invasive ductal (8500/3, 8503/3, 8521/3, 8525/3), invasive lobular (8520/3), invasive ductal and lobular mixed (8522-8524/3, 8541/3) carcinomas, and cancers of other or unspecified (8000/3-8004/3) histologic type based on the 3^rd^ edition of the International Classification of Diseases for Oncology (ICD-O-3) [13]. All patients with FBC were followed from the date of diagnosis until detection of a second primary cancer in the contralateral breast, death, loss to follow-up or until 31 December 2007, whichever of these events came first. Invasive primary cancers occurring six months after diagnosis of the FBC were defined as metachronous tumors. Cancers occurring earlier than six months were considered as synchronous tumors and were excluded from analyses. Information on human epidermal growth factor receptor 2 (HER2) amplification, stage at time of diagnosis and treatment was too frequently missing for a meaningful data analysis.

Table 1 presents an overview of the analyzed FBC cases in the registries. The proportion of histological verification of FBC was generally high with a range of 92.5 to 96.8% and for cases of metachronous primary breast cancer with up to 100% confirmation. The proportion of missing HR status information (estrogen receptor (ER) or progesterone receptor (PR)) in FBC ranged between 7.0 and 15.1%. SL did not routinely collect HR status of CBC, which was reflected in a greater proportion of missing information. Pooling three registries’ data, the median follow-up period for FBC was approximately just three years. The cohort yielded a total of 180,768 person-years of observation. We defined the following categories of HR subtype: positive (ER+ PR+), negative (ER-PR-) and mixed (ER+ PR- or ER-PR+).

**Table 1:** **Baseline characteristics of invasive breast cancer of analyzed cancer registries in Germany, 1998 to 2007**

	**Brandenburg**	**Munich**	**Saarland**	**Pooled**
**Registered cases of invasive first primary breast cancer (n)**	15,226	26,315	8,263	49,804
Histological verification (%)	94.6	92.5	96.8	93.8
Receptor status information (%)				
Estrogen positive	72.1	72.6	69.0	71.9
negative	20.9	13.5	16.0	16.2
missing	7.0	13.9	15.0	11.9
Progesterone positive	66.6	68.8	61.0	66.9
negative	26.1	17.0	23.9	20.9
missing	7.3	14.2	15.1	12.2
Median (IQR) age at diagnosis of first primary breast cancer	63 (52-72)	63 (53-73)	64 (54-74)	63 (53-73)
Person-years of observation	57,108	92,247	31,413	180,768
Median (IQR) years of follow-up	3.3 (1.3-5.9)	3.0 (1.1-5.4)	3.4 (1.3-6.0)	3.1 (1.2-5.7)
**Registered cases of metachronous contralateral breast cancer (n)**	185	287	122	594
Histological verification (%)	98.4	100.0	99.2	99.3
Receptor status information (%)				
Estrogen positive	53.0	61.0	37.7	53.7
negative	42.7	27.2	19.7	30.5
missing	4.3	11.8	42.6	15.8
Progesterone positive	44.3	53.3	27.9	45.3
negative	51.4	34.8	29.5	38.9
missing	4.3	11.8	42.6	15.8

IQR: interquartile range.

### Statistical methods

As simulation studies previously showed that analyses using completed data sets derived from multiple imputation tend to provide less biased estimates compared to complete case analyses, we used multiple imputation of HR status to account for missing data [14,15]. We assumed missingness at random and included date of diagnosis, date of birth, duration of follow-up, region code and diagnosis confirmation as additional clinical items for the imputation. We imputed 20 times applying PROC MI of SAS™ (SAS Inc., Cary, NC, USA). The results from these 20 imputed data sets were summarized using Rubin’s method [16].

We estimated the standardized incidence ratios (SIRs) of metachronous primary breast cancer to quantify the relative risk of CBC among women with FBC compared to the relative risk of developing FBC in the general population. The SIR was obtained as the ratio of the number of observed cases (O) to the number of expected cases (E). E was calculated by multiplying accumulated person-years at risk after FBC and cancer incidence rates specific for sex (female), age (0 to 4, 5 to 9, …, 80 to 84, 85+ years), five-year calendar period and the respective registry. Corresponding 95% confidence intervals (95%CIs) were based on the Poisson distribution. To obtain more precise SIR estimates of metachronous primary breast cancers, we pooled the case files and corresponding person-years at risk of the registries.

## Results

A total of 594 new CBCs among 49,804 women diagnosed with FBC were registered in the three populations from 1998 through 2007. The SIRs of CBC among women with FBC of any HR subtype ranged from 1.0 in MCR to 1.5 in BB (Table 2). In all three registries, among women with HR-positive FBC, risk of HR-positive CBC was lower compared to the risk of developing FBC in the general population. Conversely, those with HR-negative FBC had in particular an increased risk of HR-negative CBC. SIR estimates for HR-mixed second primary after HR-mixed FBC were elevated in all three registries. Table 2 shows subtype-specific SIRs for the registries that include at least five cases in total. In sensitivity analyses, where we only distinguished between ER-positive and ER-negative regardless of the PR subtype or excluded cases with missing HR status to assess whether imputation affected SIRs, estimates were similar (Table S1 and Table S2 in Additional file 1).

**Table 2:** **Standardized incidence ratios of metachronous contralateral breast cancer stratified by hormone receptor (HR)-status in Brandenburg, Munich and Saarland, 1998 to 2007**

	**Brandenburg**			**Munich**			**Saarland**			**Pooled**		
	**O**	**SIR**	**95%CI**	**O**	**SIR**	**95%CI**	**O**	**SIR**	**95%CI**	**O**	**SIR**	**95%CI**
**Any first primary**	15,226			26,315			8,263			49,804		
Any second primary	185	1.5	1.3-1.7	287	1.0	0.9-1.1	122	1.4	1.2-1.7	594	1.2	1.1-1.3
Second primary HR-positive	71	0.9	0.7-1.2	137	0.8	0.6-0.9	33	1.1	0.9-1.4	241	0.9	0.8-1.0
Second primary HR-negative	68	3.3	2.6-4.2	62	2.1	1.6-2.7	23	2.3	1.5-3.2	153	2.5	2.1-2.9
Second primary HR-mixed	38	1.9	1.4-2.6	54	1.8	1.3-2.3	14	2.0	1.2-2.9	106	2.0	1.7-2.4
**First primary HR-positive**	9,698			17,286			4,880			31,864		
Any second primary	97	1.1	0.9-1.4	149	0.8	0.6-0.9	53	1.0	0.8-1.4	299	0.9	0.8-1.0
Second primary HR-positive	44	0.8	0.6-1.0	94	0.6	0.5-0.7	26	0.9	0.6-1.2	164	0.7	0.6-0.8
Second primary HR-negative	31	2.1	1.4-3.0	17	0.8	0.5-1.2	2			50	1.1	0.8-1.4
Second primary HR-mixed	19	1.3	0.8-2.0	24	1.0	0.6-1.5	6	1.1	0.5-2.1	49	1.2	0.9-1.6
**First primary HR-negative**	2,731			2,758			1,164			6,653		
Any second primary	61	3.0	2.3-3.9	69	2.7	2.1-3.4	34	3.2	2.2-4.5	164	2.8	2.4-3.3
Second primary HR-positive	20	1.6	1.0-2.4	17	1.0	0.6-1.5	2			39	1.1	0.8-1.5
Second primary HR-negative	31	8.5	5.8-12.0	30	8.9	6.1-12.7	18	9.6	5.8-14.9	79	8.9	7.1-11.1
Second primary HR-mixed	7	2.4	1.0-4.8	14	4.3	2.4-7.1	3			24	3.7	2.5-5.2
**First primary HR- mixed**	1,682			2,524			969			5,175		
Any second primary	26	1.8	1.2-2.7	33	1.2	0.8-1.7	17	1.4	0.8-2.3	76	1.4	1.1-1.8
Second primary HR-positive	7	0.8	0.3-1.6	9	0.5	0.3-0.9	1			17	0.6	0.4-0.9
Second primary HR-negative	6	2.5	0.9-5.4	8	2.5	1.1-4.8	2			16	2.2	1.3-3.5
Second primary HR-mixed	12	5.0	2.6-8.7	11	3.2	1.7-5.7	4			27	4.2	2.8-5.9

O: observed number of cases; SIR: standardized incidence ratio; 95%CI: 95% confidence interval; HR, hormone receptor.

Pooling three registries’ data, the SIR of HR-positive CBC was 0.9 (95%CI: 0.8 to 1.0). Particularly, among women with HR-positive FBC the SIR was 0.7 (95%CI: 0.6 to 0.8). In contrast, women with FBC of any HR subtype showed an increased estimate of HR-negative CBC (SIR: 2.5; 95%CI: 2.1 to 2.9). Especially, an almost ninefold markedly elevated incidence of CBC was observed when FBC was HR-negative (SIR: 8.9; 95%CI: 7.1 to 11.1). Among patients with HR-mixed FBC risk of HR-mixed CBC was increased (SIR: 4.2; 95%CI: 2.8 to 5.9). Considering a greater proportion of missing HR status information of CBC in SL, we restricted the pooling to BB and MCR for sensitivity. In this analysis, estimates remained similar (results not shown).

According to the HR status and age at first cancer diagnosis (Table 3), for women aged lower than 50 years with HR-positive FBC, SIR of HR-positive CBC was 1.7 (95%CI: 1.2 to 2.5) while in women aged 50 years and older that risk was lower (SIR: 0.6; 95%CI: 0.5 to 0.7) compared to the risk of developing FBC in the general population. We observed exceedingly pronounced risks of CBC for women with HR-negative FBC diagnosed before the age of 50 years (SIR: 9.2; 95%CI: 7.1 to 11.9). Regarding these patients, the risk of HR-negative CBC was approximately 10 times higher than that one of HR-positive CBC.

**Table 3:** **Standardized incidence ratios of metachronous contralateral breast cancer stratified by hormone receptor (HR)-status and age in Brandenburg, Munich and Saarland overall, 1998 to 2007**

	**Women with first primary age <50**			**Women with first primary age ≥50**		
	**O**	**SIR**	**95%CI**	**O**	**SIR**	**95%CI**
**Any first primary**	9,684			40,120		
Any second primary	144	4.0	3.4-4.7	450	1.0	0.9-1.1
**First primary HR-positive**	6,129			25,735		
Any second primary	51	2.2	1.7-2.9	248	0.8	0.7-0.9
Second primary HR-positive	25	1.7	1.2-2.5	139	0.6	0.5-0.7
Second primary HR-negative	7	1.8	0.8-3.5	43	1.0	0.8-1.4
Second primary HR-mixed	11	4.8	2.5-8.4	38	1.0	0.7-1.4
**First primary HR-negative**	1,895			4,758		
Any second primary	60	9.2	7.1-11.9	104	2.0	1.7-2.5
Second primary HR-positive	8	2.6	1.4-4.5	31	0.9	0.7-1.3
Second primary HR-negative	33	25.4	17.6-35.4	46	6.1	4.5-8.1
Second primary HR-mixed	12	18.1	9.6-31.1	12	2.3	1.3-3.7

O: observed number of cases; SIR: standardized incidence ratio; 95%CI: 95% confidence interval; HR, hormone receptor.

A histology-specific analysis of FBC revealed that estimates of HR-positive CBCs were similar: ductal (SIR: 0.8; 95%CI: 0.7 to 0.9); lobular (SIR: 0.9; 95%CI: 0.7 to 1.2). The estimated SIR for HR-negative CBC was higher in invasive ductal (SIR: 2.7; 95%CI: 2.3 to 3.2) than in invasive lobular (SIR: 1.6; 95%CI: 0.9 to 2.6) FBCs.

## Discussion

We showed by German population-based data that the incidence of a subsequent CBC was modified by HR status of FBC. We observed a lower SIR of HR-positive CBC among women with HR-positive FBC while in women with HR-negative FBC the SIR of HR-negative CBC was considerably increased. Among women with first primary cancer diagnosed before the age of 50 years, the estimate of developing CBC was increased, and this increase was especially pronounced in HR-negative FBC.

As reported by previous studies, the HR status of CBC can be more similar to the HR status of FBC than would be expected by chance due to host factors. That leads to the assumption that particular women are more likely to have cancers of a certain HR subtype [17,18]. The concordance in HR subtype may be the result of common genetic and non-genetic factors that influenced the development of FBC of a particular HR subtype as well as the second primary occurrence of the same subtype in the contralateral breast [19].

Our results are in accordance to previous population-based studies that reported SIRs from about 5 to 10 for developing HR-negative CBC among women with HR-negative first primary [6-8]. On the one hand, limited relative effectiveness of chemotherapy and radiotherapy, and limited understanding of relevant targets in treatment of HR-negative breast cancer may have led to that particularly increased risk [20]. On the other hand, genetic and non-genetic factors causing HR-negative primaries may be relatively stronger compared to host factors causing HR-positive primaries.

With reference to previously published studies, results of developing HR-positive CBC after HR-positive FBC remain contradictory. While Kurian *et al*. and Sandberg *et al*. reported increased SIRs, the study of Bouchardy *et al*. and ours observed decreased SIRs [6-8]. That decrease may reasonably be linked to adjuvant hormonal treatment used among women with HR-positive FBC. It is well known that hormone treatment with tamoxifen reduces the risk of CBC [4,21,22]. This protective effect may affect cells with carcinogenic potential in the contralateral breast. Tamoxifen treatment was introduced in German guidelines in 1995. An unselected retrospective cohort study including 2,600 breast cancer patients with universal coverage in the catchment area of the Federal State of Baden-Württemberg over a 13-year observation period (1992 to 2005) reported that around 90% of endocrine-responsive patients received an adjuvant hormonal treatment since 1999 [23]. Our observations provide no strong evidence that tamoxifen treatment may increase the risk of HR-negative CBC after HR-positive FBC [5]. The increased SIR of developing HR-positive CBC after HR-positive FBC reported by Kurian *et al*. may reflect the disparities in the ability to afford hormone treatment of different ethnic groups in the U.S. [6]. The result of Sandberg *et al*. analyzing the period 1976 to 2005 may be due to the established use of tamoxifen treatment only in the last decade of observation [8].

In this study, approximately 10% of FBC had mixed HR status. Women with HR-mixed FBC showed an increased risk of CBC overall, and especially of HR-mixed CBC. Although hormone treatment with tamoxifen is used to treat both subtypes (ER+ PR- and ER-PR+), it tends to be less effective for ER-PR+. ER status seems to be the only factor importantly predictive for reductions of breast cancer recurrence and death [24]. Furthermore, Arpino *et al*. assumed that lack of PR in ER-positive breast cancer may be an indicator of abnormal growth factor signaling, which could add to resistance in tamoxifen treatment. They noticed a poorer survival in tamoxifen-treated women with ER+ PR- than in such women with ER+ PR+. Our finding of an increased risk of CBC after HR-mixed FBC may implicate those aspects [25].

Early age at first diagnosis is a well-known risk factor for CBC [2]. It has been also reported in the context of a positive family history of breast cancer and among BRCA1 and BRCA2 mutation carriers [26,27]. This suggests that more genetic than non-genetic factors may contribute to these cases and women may be more susceptible to cancer developing in both breasts. In particular, we showed a markedly increased risk of HR-negative CBC in women with age lower than 50 years, which is in line with reports that observed that the majority of BRCA-associated breast cancers were HR-negative and concordant in first and second primary [28-30].

The pooling of the cancer registries’ data enabled us to estimate SIRs of CBC in detail. Nevertheless, there are factors limiting the interpretation of our results. First, although this study provides the largest number of FBC patients at risk for CBC by HR status in Europe, we still suffer from small numbers of CBC, especially in detailed analysis by HR subtype. Second, the median years of follow-up of about three years seems to be short. However, previous studies showed no effect for time since primary diagnosis on risk for CBC by HR status [6,8]. Third, there is a lack of stage and treatment information, particularly to assess the influence of adjuvant hormonal treatment on risk of developing CBC after HR-positive FBC. However, we assume that almost all women with HR-positive FBC received adjuvant hormone treatment based on the introduction of treatment with tamoxifen in German guidelines in 1995. Available German study results underline this assumption [23]. Finally, misclassification of HR status by differences in for example tissue fixation, choice of antibody, scoring method and type of assay could have affected SIRs. Several reports documented an interobserver and interlaboratory variation for radioimmunoassay and immunohistochemical assay [31,32]. The HR status of the analyzed cases was predominantly determined by immunostains.

## Conclusions

HR status and age at diagnosis of breast cancer are modifiers for risk of CBC. Women with HR-negative FBC have an increased risk of all HR subtypes in CBC, and specifically of HR-negative. Our findings support, among these women with age lower than 50 years at FBC diagnosis, the recommendation of intensive follow-up and surveillance for secondary prevention of cancer in the contralateral breast.

## Additional file

Additional file 1:
**Pooling Brandenburg, Munich and Saarland, 1998-2007.** **Table S1.** Shows standardized incidence ratios of metachronous contralateral breast cancer stratified by estrogen receptor (ER)-status. **Table S2.** Shows standardized incidence ratios of metachronous contralateral breast cancer stratified by hormone receptor (HR)-status excluding cases with missing HR status.

## Abbreviations

95%CI: 95% confidence interval
BB: cancer registry of the Federal State of Brandenburg
CBC: contralateral breast cancer
E: number of expected cases
ER: estrogen receptor
FBC: first primary breast cancer
HER2: human epidermal growth factor receptor 2
HR: hormone receptor
MCR: Munich Cancer Registry
O: number of observed cases
PR: progesterone receptor
SIR: standardized incidence ratio
SL: Saarland Cancer Registry
