# Supplementary material for: Risk of contralateral second primary breast cancer according to hormone receptor status in Germany
Source: Breast Cancer Res. 2014 Oct 3;16(5):452. doi: 10.1186/s13058-014-0452-4 (PMC4303131; doi:10.1186/s13058-014-0452-4)
Supplement: Additional file 1: — Pooling Brandenburg, Munich and Saarland, 1998-2007. Table S1. Shows standardized incidence ratios of metachronous contralateral breast cancer stratified by estrogen receptor (ER)-status. Table S2. Shows standardized incidence ratios of metachronous contralateral breast cancer stratified by hormone receptor (HR)-status excluding cases with missing HR status. [file 13058_2014_452_MOESM1_ESM.doc]

| **Additional file 1: Table S1**: Standardized incidence ratios of metachronous contralateral breast cancer stratified by estrogen receptor (ER)-status pooling Brandenburg, Munich and Saarland,  1998-2007 | | | |
| --- | --- | --- | --- |
|  |  |  |  |
|  | **O** | **SIR** | **95%CI** |
|  |  |  |  |
| **Any first primary** | 49,804 |  |  |
| Any second primary | 594 | 1.2 | 1.1-1.3 |
| Second primary ER-positive | 322 | 1.0 | 0.9-1.1 |
| Second primary ER-negative | 181 | 2.4 | 2.1-2.8 |
|  |  |  |  |
| **First primary ER-positive** | 35,788 |  |  |
| Any second primary | 360 | 0.9 | 0.8-1.0 |
| Second primary ER-positive | 243 | 0.8 | 0.7-0.9 |
| Second primary ER-negative | 74 | 1.2 | 0.9-1.5 |
|  |  |  |  |
| **First primary ER-negative** | 8,066 |  |  |
| Any second primary | 182 | 2.4 | 2.0-2.7 |
| Second primary ER-positive | 56 | 1.2 | 0.9-1.5 |
| Second primary ER-negative | 97 | 7.6 | 6.2-9.2 |
|  |  |  |  |
| O: Observed number of cases; SIR: Standardized incidence ratio; 95%CI: 95% confidence interval | | | |

| **Additional file 1: Table S2**: Standardized incidence ratios of metachronous contralateral breast cancer stratified by hormone receptor (HR)-status^1^ pooling Brandenburg, Munich and Saarland,  1998-2007 | | | |
| --- | --- | --- | --- |
|  |  |  |  |
|  | **O** | **SIR** | **95%CI** |
|  |  |  |  |
| **Any first primary** |  |  |  |
| Any second primary | 594 | 1.2 | 1.1-1.3 |
| Second primary HR-positive | 241 | 0.8 | 0.7-0.9 |
| Second primary HR-negative | 153 | 2.6 | 2.2-3.0 |
| Second primary HR-mixed | 106 | 2.1 | 1.7-2.5 |
|  |  |  |  |
| **First primary HR-positive** |  |  |  |
| Any second primary | 299 | 0.9 | 0.8-1.0 |
| Second primary HR-positive | 164 | 0.7 | 0.6-0.9 |
| Second primary HR-negative | 50 | 1.2 | 0.9-1.6 |
| Second primary HR-mixed | 49 | 1.4 | 1.0-1.9 |
|  |  |  |  |
| **First primary HR-negative** |  |  |  |
| Any second primary | 164 | 2.8 | 2.4-3.3 |
| Second primary HR-positive | 39 | 1.0 | 0.7-1.4 |
| Second primary HR-negative | 79 | 10.5 | 8.3-13.1 |
| Second primary HR-mixed | 24 | 3.9 | 2.5-5.8 |
|  |  |  |  |
| **First primary HR- mixed** |  |  |  |
| Any second primary | 76 | 1.4 | 1.1-1.8 |
| Second primary HR-positive | 17 | 0.5 | 0.3-0.8 |
| Second primary HR-negative | 16 | 2.5 | 1.4-4.0 |
| Second primary HR-mixed | 27 | 4.8 | 3.2-7.0 |
|  |  |  |  |
| ^1^: Cases of invasive first primary breast cancer or contralateral breast cancer with missing HR-status were excluded; O: Observed number of cases; SIR: Standardized incidence ratio; 95%CI: 95% confidence interval | | | |
